# Supplementary material for: Neural manifolds for odor-driven innate and acquired appetitive preferences
Source: Nat Commun. 2023 Aug 5;14:4719. doi: 10.1038/s41467-023-40443-2 (PMC10404252; doi:10.1038/s41467-023-40443-2)
Supplement: Supplementary file 3 — Reporting Summary [file 41467_2023_40443_MOESM3_ESM.pdf]

nature portfolio

Corresponding author(s): Baranidharan Raman

Last updated by author(s): 07/15/2003

Reporting Summary

Nature Portfolio wishes to improve the reproducibility of the work that we publish. This form provides structure for consistency and transparency in reporting. For further information on Nature Portfolio policies, see our Editorial Policies and the Editorial Policy Checklist.

Statistics

For all statistical analyses, confirm that the following items are present in the figure legend, table legend, main text, or Methods section.

n/a	Confirmed	
☐	☒	The exact sample size (*n*) for each experimental group/condition, given as a discrete number and unit of measurement
☐	☒	A statement on whether measurements were taken from distinct samples or whether the same sample was measured repeatedly
☐	☒	The statistical test(s) used AND whether they are one- or two-sided<br>*Only common tests should be described solely by name; describe more complex techniques in the Methods section.*
☐	☒	A description of all covariates tested
☐	☒	A description of any assumptions or corrections, such as tests of normality and adjustment for multiple comparisons
☐	☒	A full description of the statistical parameters including central tendency (e.g. means) or other basic estimates (e.g. regression coefficient) AND variation (e.g. standard deviation) or associated estimates of uncertainty (e.g. confidence intervals)
☐	☒	For null hypothesis testing, the test statistic (e.g. F, t, r) with confidence intervals, effect sizes, degrees of freedom and P value noted<br>*Give P values as exact values whenever suitable.*
☒	☐	For Bayesian analysis, information on the choice of priors and Markov chain Monte Carlo settings
☒	☐	For hierarchical and complex designs, identification of the appropriate level for tests and full reporting of outcomes
☒	☐	Estimates of effect sizes (e.g. Cohen's *d*, Pearson's *r*), indicating how they were calculated

Our web collection on statistics for biologists contains articles on many of the points above.

Software and code

Policy information about availability of computer code

Data collection	MATLAB (2018 or newer; Mathworks Inc.), LabView 2012 (National Instruments)
Data analysis	MATLAB (2018 or newer; Mathworks Inc.), Python. All raw data and code have been made available via FigShare.

For manuscripts utilizing custom algorithms or software that are central to the research but not yet described in published literature, software must be made available to editors and reviewers. We strongly encourage code deposition in a community repository (e.g. GitHub). See the Nature Portfolio guidelines for submitting code & software for further information.

Data

Policy information about availability of data

All manuscripts must include a data availability statement. This statement should provide the following information, where applicable:

- Accession codes, unique identifiers, or web links for publicly available datasets
- A description of any restrictions on data availability
- For clinical datasets or third party data, please ensure that the statement adheres to our policy

All data presented in this paper are publicly available in Figshare (DOI: https://doi.org/10.6084/m9.figshare.22656154)

Datasets include: innate behavioral palp-opening response (POR) data, classical conditioning behavioral POR data, electrophysiological recordings of projection neurons from locust antennal lobe, chemical information about odorants used in the assay (vapor pressure, IR spectrum, NMR spectrum).

All data sets are present in raw form in the FigShare link above. All relevant code to analyze and generate final figures are also included in the FigShare. Data and code are provided in separate folders for each Main and Supplementary Figure in the manuscript.

Research involving human participants, their data, or biological material

Policy information about studies with human participants or human data. See also policy information about sex, gender (identity/presentation), and sexual orientation and race, ethnicity and racism.

Reporting on sex and gender	Our findings apply to both sexes of locusts (Schistocera americana). Throughout all the results reported, we have pooled and analyzed results from male and female locusts. In Supplementary Figure 1, we have presented an analysis comparing male and female locusts where results show these sexes behave very similarly in the scope of this study. Hence, all the experiments were performed without segregating for sex.
Reporting on race, ethnicity, or other socially relevant groupings	This is not applicable as the study was performed on locusts (Schistocerca americana).
Population characteristics	This is not applicable as the study was performed on locusts (Schistocerca americana).
Recruitment	This is not applicable as the study was performed on locusts (Schistocerca americana).
Ethics oversight	This is not applicable as the study was performed on locusts (Schistocerca americana).

Note that full information on the approval of the study protocol must also be provided in the manuscript.

Field-specific reporting

Please select the one below that is the best fit for your research. If you are not sure, read the appropriate sections before making your selection.

☒ Life sciences ☐ Behavioural & social sciences ☐ Ecological, evolutionary & environmental sciences

For a reference copy of the document with all sections, see nature.com/documents/nr-reporting-summary-flat.pdf

Life sciences study design

All studies must disclose on these points even when the disclosure is negative.

Sample size	Sample size for behavioral data in Figure 1 was validated using Monte Carlo simulations and is shown in Figure 1e. The number of projection neurons comprise —10% of the locust antenna! lobe and have previously been shown to be sufficient to capture population trends (Saha et al., 2013). The number of locusts used in classical conditioning experiments was similar to previously published literature (Nizampatnam et al., 2018, 2022).
Data exclusions	We discarded data from 42 projection neurons using a statistical test to quantify the stability of neurons across the four hours of the experiment. These 42 neurons were either lost during the experiment or their baseline firing activity was altered beyond a threshold as described in the Methods section. We recorded from 131 PNs, retained 89 PNs, and discard 42 PNs.
Replication	We have not replicated the results reported in this study. Some of the classical conditioning paradigms used in this study have been previously published in locusts and produced similar results as we report here (Nizampatnam et al., 2018, 2022).
Randomization	For the innate preferences assay, each locust was provided a different pseudorandomized sequence of the twenty-two odorants. For electrophysiology experiments, each experiment had a different pseudorandomized sequence of the twenty-two odorants. For classical conditioning, the test phase used a different pseudorandomized sequence of the four odorants for every locust.
Blinding	For innate and learned behavior assays, the experimenter was blind to the group (male or female) of locusts they were working with. To analyze innate preferences of locusts, we scored the locust behaviors with a blind approach. The scorer was only provided the videos of the locust behavior with no stimulus information. The stimulus information and scores were combined after all trials were scored.<br><br>For electrophysiological experiments, the experimenter was blind to the group (male or female) of the locusts. The neurons recorded from were random in each locust, given the recording probe was randomly placed in different regions of the antennal lobe in each experiment to record from subsets of projection neurons.

Reporting for specific materials, systems and methods

We require information from authors about some types of materials, experimental systems and methods used in many studies. Here, indicate whether each material, system or method listed is relevant to your study. If you are not sure if a list item applies to your research, read the appropriate section before selecting a response.

Materials & experimental systems

n/a	Involved in the study	
☒	☐	Antibodies
☒	☐	Eukaryotic cell lines
☒	☐	Palaeontology and archaeology
☐	☒	Animals and other organisms
☒	☐	Clinical data
☒	☐	Dual use research of concern
☒	☐	Plants

Methods

n/a	Involved in the study	
☒	☐	ChIP-seq
☒	☐	Flow cytometry
☒	☐	MRI-based neuroimaging

Animals and other research organisms

Policy information about studies involving animals; ARRIVE guidelines recommended for reporting animal research, and Sex and Gender in Research

Laboratory animals	We used post fifth-instar (young adult) locusts (Schistocerca americana) of both sexes (males and females) for all experiments in this study. These locusts were reared in a crowded in-house colony.
Wild animals	Study did not involve wild animals.
Reporting on sex	Findings apply to both sexes of locusts (Schistocerca americana)
Field-collected samples	Study did not involve field-collected samples
Ethics oversight	No ethical approval was obtained (or was necessary as the model system used are invertebrates)

Note that full information on the approval of the study protocol must also be provided in the manuscript.
