# Supplementary material for: Neural manifolds for odor-driven innate and acquired appetitive preferences
Source: Nat Commun. 2023 Aug 5;14:4719. doi: 10.1038/s41467-023-40443-2 (PMC10404252; doi:10.1038/s41467-023-40443-2)
Supplement: Supplementary file 4 — Source Data [file 41467_2023_40443_MOESM4_ESM.zip › Requirements and Instructions.rtf]

To replicate the figures presented in this study, the following software are required:
1. MATLAB (MathWorks Inc.) (2018 or newer)
2. Python 3
3. Adobe Illustrator 

Installation:
Please follow standard install instructions that accompany the software.

System requirements:
Our results were generated on a Windows operating system. (Windows 10)

Runtime:
All code sections should run in < 2 seconds. 

How to run:
The codes can simply be run section by section to produce different figure panels

Expected Output:
All analytical figure panels should be generated on the coding software. The figures were then made publication-ready using Adobe Illustrator.
