# Supplementary material for: Neural manifolds for odor-driven innate and acquired appetitive preferences
Source: Nat Commun. 2023 Aug 5;14:4719. doi: 10.1038/s41467-023-40443-2 (PMC10404252; doi:10.1038/s41467-023-40443-2)
Supplement: Supplementary file 4 — Source Data [file 41467_2023_40443_MOESM4_ESM.zip › SupplementaryFigure5/readMe.rtf]

Code for this figure is included in scripts for main Figure 9
